# A Transfer-Learning-Based Deep Convolutional Neural Network for Predicting Leukemia-Related Phosphorylation Sites from Protein Primary Sequences

**DOI:** 10.3390/ijms23031741

**Published:** 2022-02-03

**Authors:** Jian He, Yanling Wu, Xuemei Pu, Menglong Li, Yanzhi Guo

**Affiliations:** College of Chemistry, Sichuan University, Chengdu 610064, China; hejian@stu.scu.edu.cn (J.H.); wuyanling@stu.scu.edu.cn (Y.W.); xmpuscu@scu.edu.cn (X.P.)

**Keywords:** leukemia, protein phosphorylation site, protein primary sequences, machine-learning, deep-learning, transfer-learning

## Abstract

As one of the most important post-translational modifications (PTMs), phosphorylation refers to the binding of a phosphate group with amino acid residues like Ser (S), Thr (T) and Tyr (Y) thus resulting in diverse functions at the molecular level. Abnormal phosphorylation has been proved to be closely related with human diseases. To our knowledge, no research has been reported describing specific disease-associated phosphorylation sites prediction which is of great significance for comprehensive understanding of disease mechanism. In this work, focusing on three types of leukemia, we aim to develop a reliable leukemia-related phosphorylation site prediction models by combing deep convolutional neural network (CNN) with transfer-learning. CNN could automatically discover complex representations of phosphorylation patterns from the raw sequences, and hence it provides a powerful tool for improvement of leukemia-related phosphorylation site prediction. With the largest dataset of myelogenous leukemia, the optimal models for S/T/Y phosphorylation sites give the AUC values of 0.8784, 0.8328 and 0.7716 respectively. When transferred learning on the small size datasets, the models for T-cell and lymphoid leukemia also give the promising performance by common sharing the optimal parameters. Compared with other five machine-learning methods, our CNN models reveal the superior performance. Finally, the leukemia-related pathogenesis analysis and distribution analysis on phosphorylated proteins along with K-means clustering analysis and position-specific conversation profiles on the phosphorylation site all indicate the strong practical feasibility of our easy-to-use CNN models.

## 1. Introduction

Post-translational modifications (PTMs) of proteins are a pivotal mechanism regulating cellular functions by the covalent and generally enzymatic modification, which plays vital roles in regulating various biological processes [1]. Protein phosphorylation is one of the most important posttranslational modifications in eukaryotes [2]. By covalently attaching phosphate moieties to Ser (S), Thr (T) or Tyr (Y) residues in a dynamic manner [3,4], it regulates many cellular processes such as DNA growth, metabolism and cell cycle control [5,6]. Currently, a number of phosphorylation sites have been accurately verified by different experimental techniques and related databases have been built, like Database of dbPSP 2.0 [7], PhosphoPep [8] and Phospho.ELM [9].

Based on the available phosphorylation site data, the machine/deep-learning methods have been proposed for phosphorylation site prediction. Among them, traditional machine-learning models were developed by manually extracting effective features to represent phosphorylation site information, such as Shannon entropy, relative entropy, information gain, protein disordered property, the average cumulative hydrophobicity, etc. [10,11,12]. Nowadays convolutional neural network (CNN)-based deep-learning methods have also been proposed by just taking raw sequence data as input without manual feature extraction. For example, Wang et. al. [13] has used a novel two-dimensional attention mechanism to predict general and kinase-specific phosphorylation sites. Luo et. al. [14] have proposed a densely connected convolutional neuron network blocks which can capture multiple representations of sequences to make final phosphorylation prediction by intra block concatenation layers and inter block concatenation layers; Ahmed et. al. [15] used a stacked long short-term memory recurrent network which learns the protein representations from conjoint protein descriptors for predicting phosphorylation sites.

Despite the tremendous potential of the deep-learning, the challenge in developing deep-learning-based prediction methods is the requirement of large amounts of data [16]. Due to lacking enough experimental data, the reliable deep-learning models cannot be achieved on such a small size dataset. Nowadays, transfer-learning can be used to solve this problem. It refers to the strategy of migrating knowledge for a new task from a relative task that has already been learned. For example, Liu et. al. [17] have proposed a novel transfer-deep-learning method for predicting ubiquitination sites of multiple species.

It has been confirmed that phosphorylation sites are associated with altered protein functions [18] and abnormal phosphorylation has now been proved to be closely related with human diseases [19]. Randall et. al. [20] have shown that the increased phosphorylation of TDP-43 leads to a number of neurotoxic effects including reduced liquid liquid phase separation dynamicity, changes in splicing, cytoplasmic mislocalization and aggregation, which ultimately contributes to neurotoxicity and neurodegeneration and may cause amyotrophic lateral sclerosis and frontotemporal lobar degeneration. Zhang et. al. [21] have declared the phosphorylation of amyloid precursor protein (APP) is one of the keys for modulating the generation of amyloid-β and phosphorylation abnormalities of APP would lead to abnormal accumulation of amyloid-β in the central nervous system, which is a hallmark of Alzheimer’s disease. Brustovetsky [22] has found that collapsin response mediator protein 2 phosphorylation inhibits the interaction between Drp1 and Miro 2 which are involved in regulating mitochondrial dynamics and leading to Huntington’s disease. Simon et. al. [23] have reported that phosphorylation at Ser289 in the CaM autoregulatory do-main by Ribosomal S6 kinases reduces the apoptotic activity of Death-associated protein kinase I which leads to neurodegenerative diseases such as ischemic stroke and Alzheimer s disease. So a number of disease-associated phosphorylation sites have been recognized and databases have been constructed, like Database of qPhos [24], PTMD [25] and PhosphoSitePlus [26]. So far, few studies are reported focusing on human disease-related phosphorylation site prediction, except the work by Xu et. al. [27] who have proposed a combined feature selection method-based SVM that incorporates mRMR filtering process and forward feature selection process to identify disease-related phosphorylation sites. Nowadays, there is no research for specific disease-associated phosphorylation sites prediction. So, it is urgent to identify such specific disease-associated phosphorylation sites in large-scale phosphoproteome, which may provide a guide for the comprehensive understanding of disease mechanism as well as biomedical drug design [28].

As we know, leukemia is a common blood malignant tumor caused by the anomalous growth of leukocytes and diseased leukemia cells can enter blood to affect the metabolism of normal cells in the body [29]. Leukemia is a systemic infectious malignant tumor and the viral expression process is closely related to protein phosphorylation [30,31]. Currently, leukemia-related phosphorylation site data has been accumulated. In this context, we aim to develop the reliable prediction model to distinguish the leukemia-related phosphorylation sites from the non-phosphorylation sites.

Most existing computational methods for phosphorylation site prediction are based on manual feature extraction. Existing manual feature extraction techniques are directed at one-sided material information of the phosphorylation site, which cannot adequately describe the complex biological properties of phosphorylated modification sites, and this would probably result in incomplete or biased feature representation [32]. Deep learning is based on end-to-end can automatically discover complex patterns and capture the high-level abstraction adaptively from the training data, so it is more applicable to variable natural data than manual extraction and has good generalization ability and robustness, which can select the best discrimination feature subset for the final prediction model. Deep-learning as the cutting-edge method allow their computational models to be fed with raw data and automatically discover the complex representations of protein functions needed for classification, hence it provides a powerful tool for improvement of protein functions prediction.

In this work, with only one convolution, pooling and dense layer respectively, an easy-to-use CNN architecture was developed for predicting leukemia-related phosphorylation sites just using the protein primary sequences. Here, the phosphorylation site data were collected for three leukemia classes, including myelogenous, lymphocytic and T-cell leukemia. Since myelogenous has the largest dataset and could provide sufficient samples to achieve a CNN model, myelogenous-related S/T/Y phosphorylation site prediction models were constructed by our CNN architecture. To evaluate the performance and prove the advantage of this method, we also used other five machine-learning methods to construct the prediction models. Through comparisons, our CNN model yields promising performance, which are superior to the five other models, when all models are based on sequence information. Finally, the pre-models derived from myelogenous-related datasets were used to construct prediction models for lymphocytic and T-cell leukemia with the small size datasets by a deep transfer-learning framework and the good performance on small datasets proves the strong feasibility of our model.

## 2. Results and Discussion

### 2.1. Functional Analysis on the Phosphorylated Proteins

**Figure 1:**
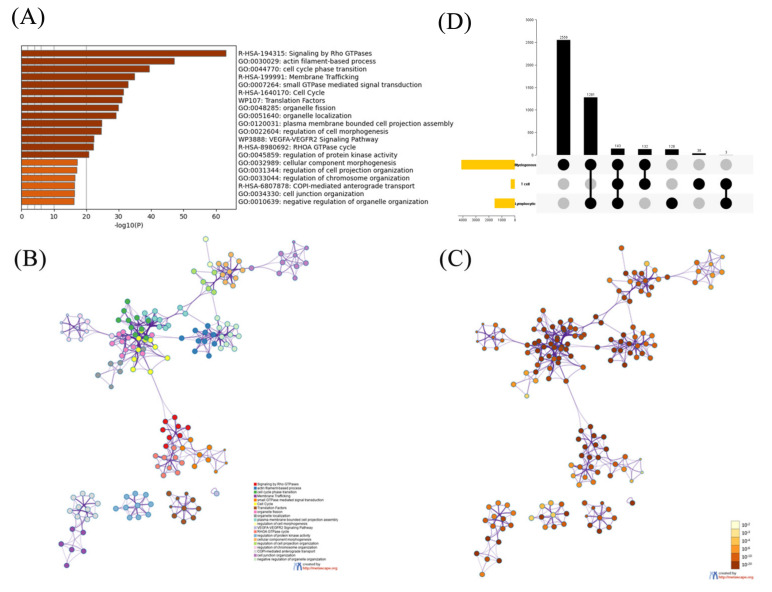
Top 20 clusters with their representative enriched terms (one per cluster) of genes which have leukemia-related phosphorylation sites. (**A**) Heatmap of *p*-value for each cluster. (**B**) The networks of enriched terms with nodes colored by cluster ID, where nodes sharing the same cluster ID are typically close to each other. The most statistically significant term within a cluster is chosen to represent the cluster. (**C**) The network with nodes colored by *p*-values, where terms containing more genes tend to have a more significant *p*-value. (**D**) Statistics on the number of different types of leukemia proteins.

In our three leukemia-related datasets, all phosphorylation sites are from 8011 phosphorylated proteins. In order to understand an in-depth knowledge about genes associated with leukemia, we performed functional pathway enrichment analysis using Metascape database [33] and 1707 genes were found in all enriched pathways. Functional pathway enrichment is a statistical analysis performed by analytical tools to mine the databases for gene function classes that have significant relevance to the biological problem we are studying. The statistical principle is to test the significance of a functional class in a set of genes (co-expressed or differentially expressed) by means of hypergeometric distribution, enrichment analysis and false positive analysis, and to identify the functional classes of genes that are significantly associated with low false positive rate and targeting. The heatmap in Figure 1A shows the most significant pathways and Figure 1B,C show the functional pathway correlation networks constructed based on gene clustering enrichment and *p*-value, respectively. Meanwhile, the detailed information of the top 20 enriched terms are listed in Supplementary Table S1. The results reveal that the pathways of these genes are mainly related to cellular morphological changes, including organelle fission, cell cycle, cell cycle phase transition and membrane trafficking, etc. In recent years, many researchers have focused on the cellular morphological changes in leukemia, indicating that cellular morphological changes play an important role in serving as biomarkers of leukemia [34,35]. We can also see that most of the genes are clustered on the right side of Figure 1B and they are all very significant according to their values from Figure 1C, so they are mainly involved in cellular morphological changes related pathways, indicating that the cellular morphological changes related pathways are important for leukemia. In addition, actin filament-based process, Signaling by Rho GTPases and small GTPase mediated signal transduction pathways are also demonstrated to be closely related to leukemia from our analysis results, since there are over 400 genes are involved in the three pathways respectively.

In addition, the distribution of the phosphorylated proteins in different types of leukemia was also given in Figure 1D. We can see that each type of leukemia commonly has their own specific proteins and few are the common proteins among them, so individual functional analysis was also performed on the corresponding proteins of each leukemia type (Supplementary Figure S1). The detailed information is shown in Supplementary Table S1. Similarly, few common enriched pathways are observed among them, except WP3888 (VEGFA-VEGFR2 Signaling Pathway) that is also enriched in Figure 1A, indicating that it may play an important role in the progression of leukemia. From these results, we can conclude that since functions of proteins with the different types of leukemia-related phosphorylation sites are different, it is of more practical significance to construct the prediction model for each specific type of leukemia.

**Figure 2:**
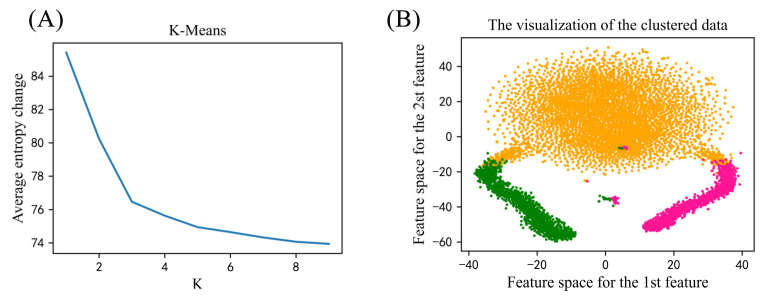
K-means clustering analysis. (**A**) Average entropy changes of different categories. (**B**) The visualization of the clustered data.

Finally, we performed K-means clustering analysis on all the phosphorylation sites of myelogenous leukemia. By using the K-means algorithm, all samples can be divided into K clusters according to the distances between them. The optimal categorical K value makes samples within one cluster be the closest connected and those across clusters having with the largest distances. Here, each phosphorylation peptide with length of 201 was transformed into a one-hot vector. As shown in Figure 2, the optimal categorical K value is 3, indicating that there is still data variability within the dataset although they are all associated with myelogenous leukemia. In addition, clustering analysis on S, T and Y datasets respectively also given the same observation that each dataset can be clearly classified into three clusters (Supplementary Figure S2). Because of the obvious variability between samples, in process of the random division of training and testing sets, in order to ensure the completeness of the data distribution, 90% of the data in each cluster were randomly extracted as training set and 10% of each cluster as the testing set. This random selection was repeated 100 times to give a more reliable evaluation of model performance.

### 2.2. The Position-Specific Conversation Profiles of the Phosphorylation Peptides

Neighboring residues are not equally important to the functional sites. Some are essential for the proper structure and function of the proteins, whereas others can be readily replaced [36]. In fact, for a given sequence fragment, the conservation varies from one position to another and some residues with high conversation might have contribution to leukemia-related phosphorylation site. So, the small-ranged amino acids around the phosphorylation sites have been considered as the primary sequence features to represent protein sequence information for PTMs sites prediction [37]. Thus, it is necessary to analyze the importance of the neighboring positions around the leukemia-related phosphorylation sites.

**Figure 3:**
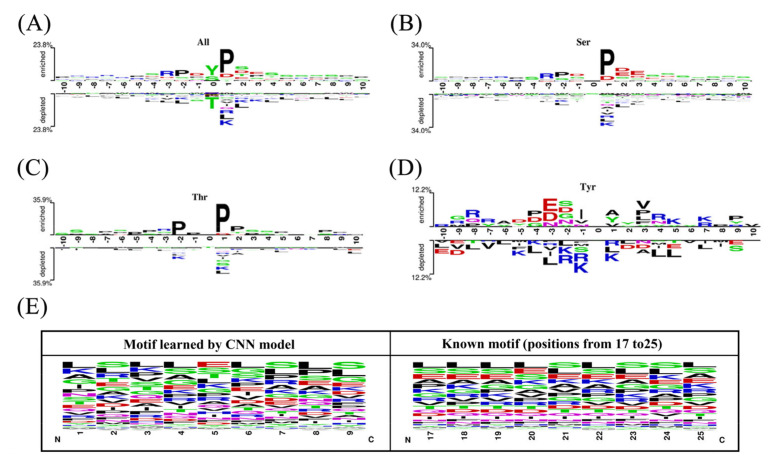
(**A**–**D**) The different position-specific distribution of amino acids between leukemia-related phosphorylation sites and non-phosphorylation sites segments for All, Ser, Thr and Tyr peptides, respectively. (**E**) The comparison between the motif learned by the deep CNN model and the known motif of leukemia-related phosphorylation sites.

The work of Nakariyakul et. al. has indicated that a protein domain is basically between ten residues [38]. Here, for the position-specific conversation analysis, we set the length of the phosphorylation segments as 21-nts, so each segment contains the middle phosphorylation sites and its flanking of 10-nts on both up- and down-stream sides. Based on the positive and negative samples, amino acids enrichment analysis was performed and the graphical sequence logo (*p* < 0.05 by *t*-test) was generated by Two Sample Logo [39]. Figure 3A–D show residue position-specific conservation differences between positive and negative sample for all sites, S, T and Y, respectively.

By means of parallel contrast, it obviously shows that distributions of the same residues have a large enough difference among flanking positions. As described by Ding et al. [40], same residues may carry different information during evolutionary history and a residue in the conserved position usually shows stronger functional relevance than one in a non-conserved position from a biological view. We found a high enrichment of Proline and Tyrosine in all phosphorylation site data and the obvious deletion in negative samples. While for Y phosphorylation sites, more glutamic acid, aspartic acid, alanine and tyrosine exist in positive samples and arginine, lysine and leucine appear more frequently in negative samples. In addition, by vertical contrast, a significant residue distribution difference can also be observed between positive and negative samples. Especially for the positive samples, proline is always more clustered after the phosphorylation sites. However, there is no significant motif pattern in the negative samples.

One of the distinctive advantages of the deep-learning model is the ability to automatically extract predictive features from inputs during the model training [41]. We explored features that were learned in our deep CNN by investigating the test sequences that activated the filters in the convolution layer. When the filter is slid over *N*-terminal sequence, it functions as motif detector and becomes activated when certain position matches its preference. By the fraction of filters activated at positions, we observed that the majority of filters were activated when they convolved a continuous region including the positions from 17 to 25 in Y dataset, the positions from 30 to 39 in S dataset and the positions from 11 to 18 in T dataset. These activation sequences were aligned to obtain the learned basis sequences represented by the position weight matrix for deep-learning. The results show that the discovered amino acid motifs revealed by deep CNN yield high-similarity shape to known motifs for three classes datasets. We used WebLogo tool [42] to generate known amino acid motif and discovered motif revealed by the CNN model of the Y dataset (Figure 3E). Though Y dataset exhibits no significant difference between phosphorylation peptides and non-phosphorylation ones by the position-specific conservation analysis, the similarity between the two motifs is very high which also further validates the feasibility of the CNN to automatically extract sequence feature information.

### 2.3. The Phosphorylation Peptide Length Optimization and Comparisons with Five Machine-Learning Methods

**Figure 4:**
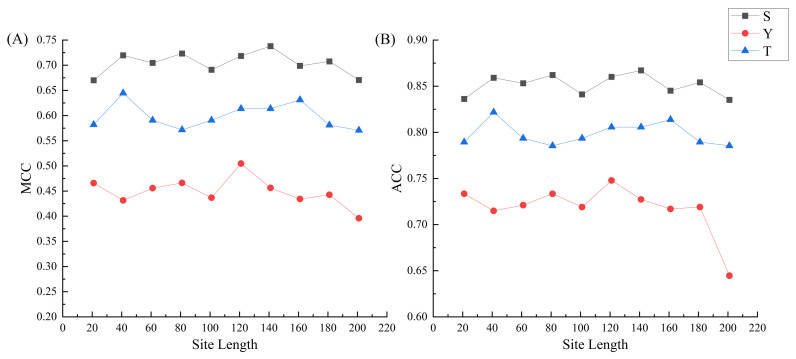
The performance of the models based on different phosphorylation peptide lengths. (**A**) The MCC values of the models on ten phosphorylation peptides lengths. (**B**) The ACC values of the models on ten phosphorylation peptides lengths.

Because of the spatially folded structures of proteins, the sequentially distant residues may be in close proximity to the variant site in the spatial structure, which affect the environmental information of the phosphorylation sites. Here, we used different phosphorylation peptide lengths to build prediction model and aimed to find the optimal peptide length for S/T/Y phosphorylation sites, respectively. In fact, 10 different segments in n-site units (n = 10, 20, …, 100) were utilized. Each fragment peptide contains intermediate phosphorylation sites and n-site elements on both upstream and downstream sides. The prediction performance of CNN models based on 10 different peptides lengths are shown in Figure 4. We found that different lengths of phosphorylation peptides do affect the performance of the models and the optimal peptide lengths for S, T and Y models are 141, 41 and 121, respectively. 

In order to demonstrate the superiority of the CNN models, based on the optimal peptide lengths for S, T and Y, other five machine-learning algorithms were also used to construct the prediction models, including support vector machine (SVM), naive Bayes (NB), K-nearest neighbors (KNN), random forest (RF) and eXtreme gradient boosting (XGBoost) since these algorithms are commonly used in studies of phosphorylation site prediction. Here, the CNN models can be implemented depending solely on protein primary sequences, requiring no prior knowledge of the variants on sequences [43]. But machine-learning methods cannot directly recognize sequences as input, so we used dictionary encoding to ensure the uniformity of sequence features. Each residue in the phosphorylation peptides is represented by an ordinal number, in which each of the 20 basic amino acids is assigned a number from 1 to 20 [44]. Thus, each peptide is represented by a one-letter code and transformed into an L-dimensional vector, where L is the length of the peptides.

**Figure 5:**
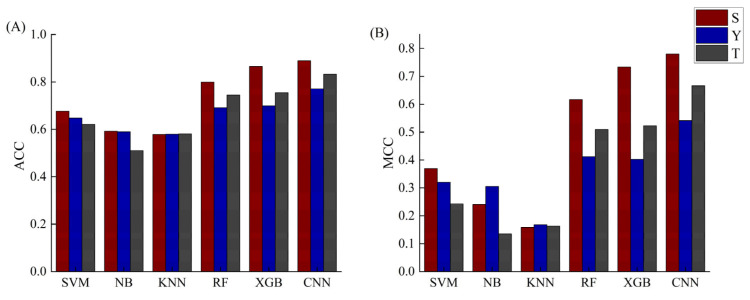
The comparisons between CNN and five machine-learning methods based on protein sequences. (**A**) The ACC values of CNN and five machine-learning methods; (**B**) The MCC values of CNN and five machine-learning methods.

In order to achieve convincing comparisons, the performance of each machine-learning model was given by the average of 100 random selections of testing sets. The comparison results of our method with other five methods are visually shown in Figure 5. All detailed prediction results of the six methods are listed in Supplementary Table S2. We know that traditional machine learning usually requires tedious feature engineering steps to obtain accurate prediction results, while CNN is an end-to-end learning that does not require hand-designed rules after data input, and CNN can learn the rules by optimizing the loss function to mine the potential features of the data as much as possible. On average, our model shows the significant improvement for predicting phosphorylation sites for the current leukemia datasets. We can see that in terms of ACC and MCC, the CNN model yields the best performance with the average prediction accuracies of 88.89%, 80.87% and 77.39% for S, T and Y, respectively.

### 2.4. Construction of the Final Predict Models

Cross-validation test can reduce the contingency caused by the single division of dataset and improve generalization ability. Based on the optimal peptide lengths, the final phosphorylation site prediction models were constructed for S, T and Y respectively. Based on the whole dataset,10-fold cross validation was used to achieve the optimal parameters of CNN models, as shown in Table 1.

**Figure 6:**
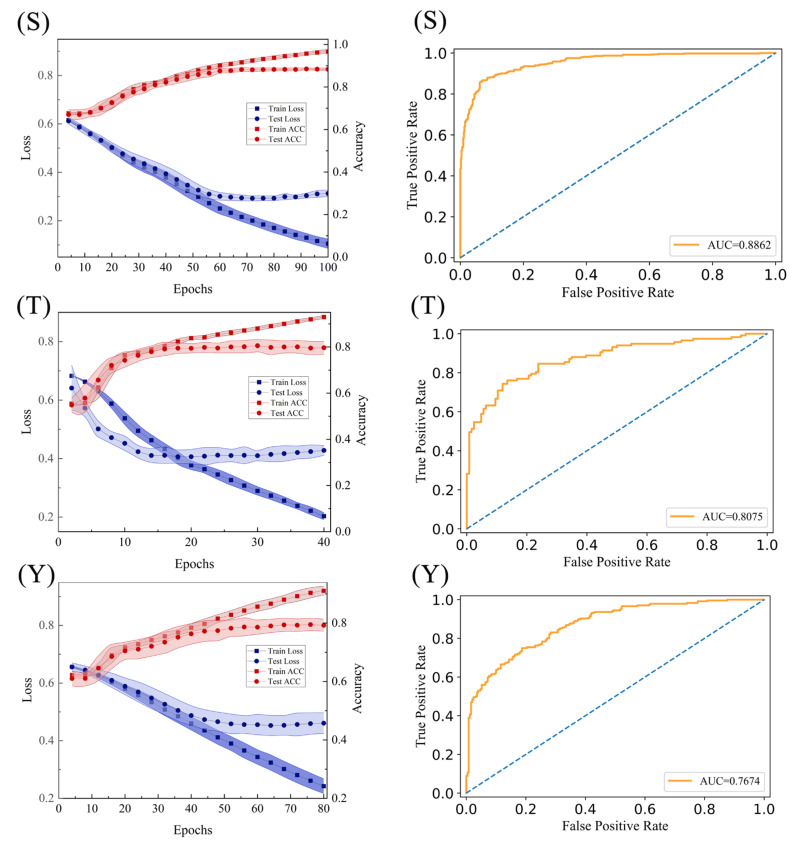
The learning curve and ROC curve of final diagnosis models. (S, T and Y) on the left column show the learning curves of the final model of S, T and Y datasets respectively; (S, T and Y) on the right column show the ROC curves of the final model of S, T and Y datasets respectively.

Table 2 shows the prediction results of the three final CNN models by 10-fold cross-validation. The proposed models still yield promising performance with ACC of 88.89%, 80.87% and 77.39% for S, T and Y models. In addition, we drew the learning curves and ROC curves of the final models, as shown in Figure 6. Through the loss curves of Figure 6 (S, T and Y) in Figure 6 on the left column, we can see that the model is reliable and does not exhibit obvious over-fitting. It can be also observed that the loss no longer decreases and the model tends to converge. Moreover, the AUC values are 0.8784, 0.8328 and 0.7716 respectively for S, T and Y models in Figure 6 on the right column, which means a satisfactory performance by the final model. In addition, it should be noted that S model with the largest dataset yields the best performance, so for model-constructing, more samples tend to give a better prediction.

Finally, the specificity of the final models was further tested. Since amount of remaining negative samples are not included in the final prediction model, they can be used as the independent negatives. The number of independent negative samples for S, T and Y are 17,207, 25,340 and 18,149, respectively and our models give the prediction accuracy of 0.8905, 0.8009 and 0.8484, respectively, which indicates that about 10–20% of them are predicted as the phosphorylation sites. However, it is reasonable that the potential phosphorylated sites from the current negatives would be probably discovered in the future.

### 2.5. Transfer-Learning on Two Other Small Size Datasets

Currently, very limited phosphorylation sites for T-cell and lymphocytic leukemia are available, so the model and the prediction parameters cannot be fully trained and optimized based on such small size datasets. Based on the large S, T and Y datasets of myelogenous, we have achieved three reliable CNN models, as shown in Table 2. Therefore, by common sharing the optimal parameters shown in Table 1, the prediction models were also constructed for T-cell and lymphocytic leukemia, respectively. Except the smallest datasets of T and Y sites of T-cell leukemia that were validated by leave-one-out testing, other models were validated by 10-fold cross validation. The average prediction results are presented in Table 3. We can see that all the six models yield the promising results. Among them, five models achieve the high SE of over 90%, indicating that for different leukemia classes, the transfer-learning based CNN could be an effective strategy for leukemia-related phosphorylation site prediction.

## 3. Materials and Methods

### 3.1. Datasets

Protein phosphorylation data for *Homo sapiens* were collected from PhosphositePlus [26]. PhosphositePlus is an open, dynamic, continuously curated and highly interactive systems biology resource for studying experimentally observed PTMs in the regulation of biological processes. From PhosphositePlus, we initially used multiple keywords of “Homo sapiens”, “phosphorylation” and “leukemia” to extract experimentally identified phosphorylation sites. In this way, 30,819 phosphorylation sites were achieved from 8011 proteins, including 27,406 myelogenous leukemia phosphorylation sites, 465 T-cell leukemia phosphorylation sites and 2944 lymphocytic leukemia phosphorylation sites, respectively.

Protein sequence information are from The UCSC Genome Browser Database [45]. Reports have shown that *N*-terminal residues appear to provide the targeting information for protein functional expression. Some effectors, e. g. PopD in *Pseudomonas aeruginosa* [46], only depends on the first ~50 residues to be secreted or trans-located and the translocation signals in some Yops are located in the first 50–100 residues [47]. Due to the spatially folded structure of proteins makes it possible for distant residues in the protein sequences to affect the environmental information of the variant site. Therefore, protein functional expression may require about 100 residues around the functional sites, so we selected peptides of 201-length with sequence which phosphorylation sites in the middle surrounded by flanking 100 residues on each side. For terminal amino acid site, where the number of flanking amino acid is less than 100, appropriate number of dummy residue ‘X’ was padded to complete the peptide. The convolutional feature map of the same size can be obtained after inputting the complementary equal-length data, which can then be input into the fully connected layer for further processing of the identified spread-out vectors to achieve the purpose of integrating information. 

CD-HIT stands for “cluster database at high identity with tolerance”. The program takes a fasta format sequence database as input and produces a set of ’non-redundant’ representative sequences as output. Since redundant sequences would lead to overfitting, we used cd-hit [48] to remove sequence redundancy. Entering the fasta format sequence files with identity threshold of 0.7, the redundant and similar sequences are removed by the method of sequence comparison and clustering and finally the non-redundant sequence files are obtained. For three leukemia classes, the details about the phosphorylation site data can be seen in Table 4.

For negative samples, we extracted the S, T and Y residues data that were not experimentally confirmed from the 8011 proteins that contain experimentally determined phosphorylation sites. After removing redundancy with the peptide length of 201, the number of negative samples is much larger than that of the positive ones, so the equivalent number of negative samples were randomly extracted.

### 3.2. Convolutional Neural Network and Transfer-Learning

Like traditional neural network architecture, CNN transmits information from the input to the output layer by layer [49]. CNN is a multi-layer perceptron that is composed of convolution layers, rectification layers, pooling layers and fully connected layers. Convolution layers are used to extract different features of input by using back-propagation algorithm and the rectification layers make the output of the convolution layer become nonlinear mapping. Then pooling layers divide the features into multiple regions and take the maximum or average value to obtain new features at small scales for preventing overfitting. Finally, fully connected layers combine local features into global features and calculate scores. By using back-propagation algorithm, convolution layers in CNNs achieve self-learning and directly extracts the features from the sequence information, which skips steps of feature extraction from the sequence objects, and feature selection for determining effective features.

Transfer-learning means the ability of a system to recognize and apply knowledge and skills learned in previous domains/tasks to novel domains/tasks, which is one of the major types in machine-learning. In general terms, transfer-learning is commonly used to find similarities between existing knowledge and new knowledge, and then use existing knowledge to learn the new knowledge [50]. Transfer- learning can be classified according to the learning method as instance-based migration, feature-based migration and shared parameter-based migration. There are various subtypes of leukemia depending on the cell type, but they are all malignant clones of hematopoietic stem cells that affect normal physiological mechanisms. Here, we adopted shared parameter-based transfer-learning by using a deep learning pre-model trained on myelogenous leukemia phosphorylation site dataset which have sufficient samples, so as to train T-cell and lymphocytic leukemia-related phosphorylation site prediction models

**Figure 7:**
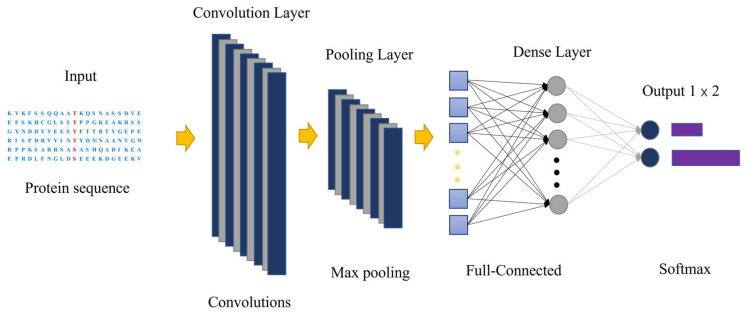
The architecture of deep convolutional neural network for leukemia-related phosphorylation sites prediction.

Our pre-model of CNN architecture is shown in Figure 7. The input is the raw sequence peptides. The number of filters in the convolutional layer is determined by the optimization results of models. After convolution layer, the rectified linear units are used to output the filter scanning results that are higher than the thresholds. Max pooling is applied in the pooling layer to reduce variance and increase translational invariance by computing the maximum value of a feature over a region. All the pooled results are merged into one vector, input into the fully connected layer and batch the vector. To avoid overfitting, a dropout layer is employed behind the fully connected layer. Finally, sigmoid function is used to predict the probability of phosphorylation sites in the output layer. Here we used S dataset as an example to illustrate how CNN architecture works. The network accepts features of proteins of 141 amino acids as input. The first convolutional layer performs 50 convolutions with 20 × 9 filter on the dictionary encoding matrix, producing 50 feature maps of size 1 × 133. The second pooling layer performs 1 × 2 spatial pooling of each feature map using the max value, producing 50 feature maps of size 1 × 66. All the pooling results are joined together into one vector by flattening. The hidden features in this vector are fully connected to a hidden layer, which are fully connected to 2 output nodes to predict the probability of leukemia-related phosphorylation site and non-leukemia-related phosphorylation site. The output node uses SoftMax function as activation function, whereas all the nodes in the other layers use rectified linear function as activation function.

After determining the pre-model, the parameter settings are shared with the small sample dataset and all layers (including convolutional layers) retrained on the small sample datasets. The new model is fine-tuned by validation method to fit the T-cell leukemia-related and lymphocytic leukemia-related phosphorylation data. In this work, we aim to use the simple model to avoid manual feature extraction, the overfitting problem caused by complex models and improve the suitability of deep- learning models for small sample datasets.

### 3.3. Traditional Machine-Learning Methods

Five traditional machine-learning methods were used to compare with our new CNN model, including RF, XGB, NB, KNN and SVM. SVM, proposed by Vapnik [51], is considered as one of the most accurate tools available. The basic idea of SVM is to find a hyperplane which separates different groups of feature vectors with a maximum margin. SVM chooses kernel trick which casts the data into a higher dimensional space where the data can be linearly separable. NB is a statistical classification method, which is a class of algorithms that uses knowledge of probability statistics for classification [52]. The core idea of KNN is to calculate the distance between the samples in the test set and all the samples in the training set according to the classification of the samples in the training set, and select the results of the first K test samples that are closest to the training samples according to the set K value, and the category in which most of the training samples are in the result is the category of this test sample [53]. RF is operated by constructing multitudes of decision trees at training time and outputting the mode of classes of individual trees [54]. The method unites bagging idea and the random selection of features in order to establish a collection of decision trees with controlled variation [55]. XGBoost is an integrated learning method with CART-based classifiers [56]. Unlike random forests that assign the same voting weight to each decision tree, the generation of the next decision tree in the XGBoost algorithm is correlated with the training and prediction of the previous decision tree (by assigning higher learning weights to samples with lower accuracy in the previous round of decision tree training to improve the model accuracy).

### 3.4. Model Validation and Evaluation

Four effective performance metrics including sensitivity (SE), specificity (SP), accuracy (ACC) and Matthew’s correlation coefficient (MCC) were used for performance assessment. SE, SP, and ACC indicate the predictive success rates on positive, negative, and overall samples, respectively. MCC accounts for true and false positives and negatives and is usually considered a balanced measure that can be used even if the classes are of very different sizes. These metrics are defined as follows:

(1) $$SE\;=\;TP/\left(TP+FN\right)$$

(2) $$SP\;=\;TN/\left(TN+FP\right)$$

(3) $$ACC\;=\;\left(TP+TN\right)/\left(TP+FP+FN+TN\right)$$

(4) $$MCC\;=\;\frac{TP\times TN-FP\times FN}{\sqrt{\left(TP+FN\right)\;\times \;\left(TP+FP\right)\;\times \;\left(TN+FP\right)\;\times \;\left(TN+FN\right)}}$$

where *TP*, *FP*, *TN* and *FN* are true positive, false positive, true negative and false negative, respectively. In addition, receiver operating characteristic (ROC) curve was also employed to assess the performance of prediction models. A ROC curve is a plot of true-positive rate (SE) versus false-positive rate (1-SP). A classifier can get a true-positive rate and a false-positive rate point pair based on its performance on the test sample, and this point pair can be mapped to a point on the ROC plane. The ROC curve is an intuitive way to indicate the performance of a classifier, and the area under the curve (AUC) is used to achieve a value to mark the performance of a classifier. AUC ranges from 0 to 1. Typically, larger AUC indicates better performance of the model, AUC values of 0.5 represents a random classifier.

## 4. Conclusions

Studies have indicated that many diseases are closely related with abnormal phosphorylation. Therefore, the large-scale identification of phosphorylation sites has potential applications in disease treatment and drug design. But existing wet-lab technologies for targeting phosphorylation sites are costly and time consuming. Thus, computational algorithms can efficiently accelerate the annotation of unknown phosphorylation sites. Numerous machine learning-based methods have been implemented for phosphorylation sites prediction. However, no method focuses on specific disease-associated phosphorylation sites prediction. We know that disease pathogenesis is specific, so we analyzed three leukemia subtypes to achieve the effective computational predictions of leukemia-related phosphorylation sites, so as to provide reference information for predicting potential phosphorylation sites and leukemia treatments.

When using machine-learning methods to solve prediction problems from biological sequences, we usually face challenges in selecting appropriate machine-learning algorithms, extracting effective features and carrying out reasonable optimization. Especially feature extraction is a key step that mainly determines the prediction performance. As a cutting-edge machine-learning method, deep learning has the ability to automatically discover complex representations of phosphorylation patterns from the raw sequences, and hence it provides a powerful tool for improvement of leukemia-related phosphorylation site prediction.

In this paper, a new transfer-learning-based deep CNN was proposed for predicting leukemia-related phosphorylation sites from protein primary sequences. Focusing on three types of leukemia, we firstly analyzed the gene pathways of the phosphorylated proteins with leukemia-related phosphorylation sites, revealing that the pathways of these genes are mainly related to cellular morphological changes, especially WP3888 (VEGFA-VEGFR2 Signaling Pathway) may play an important role in the progression of leukemia. The distribution analysis on the phosphorylated proteins in three different types of leukemia shows that there are few sharing proteins between them, so it is of practical significance to construct the prediction model for each specific type of leukemia. K-means clustering analysis on all the phosphorylation sites of myelogenous leukemia reveals the obvious variability between samples, so in process of the random division of training and testing sets, samples in all clusters should be partly extracted to ensure the completeness of the data distribution. Meanwhile, the position-specific conservation difference analysis was performed between positive and negative sample for all, S, T and Y sites respectively and the amino acid motifs revealed by CNN have a very high-similarity shape to the known motifs which validates the feasibility of the CNN to automatically extract sequence feature information.

Based on the largest S/T/Y datasets of myelogenous-related phosphorylation sites, the optimal peptide lengths for S, T and Y models were achieved and they are 141, 41 and 121, respectively. By comparing with other five machine-learning methods, CNN yields the best performance on 100 times testing. By 10-fold cross validation, the final models for S/T/Y phosphorylation sites were achieved and give the AUC values of 0.8784, 0.8328 and 0.7716 for S, T and Y, respectively. Finally, the optimal parameter of the final S/T/Y myelogenous-related models were transferred to construct the prediction models for the small datasets of lymphocytic and T-cell. The satisfactory prediction results prove that the transfer-learning based CNN is an effective strategy for leukemia-related phosphorylation site prediction.

We expect that with the accumulation of newly discovered leukemia-related phosphorylation sites data, the models constructed in this work can be further verified and will be a useful supplementary tool for identifying leukemia-related phosphorylation sites.

## Figures and Tables

**Table 1:** The optimal parameters of CNN models by 10-fold cross validation.

Phosphorylation	Length	Kernel_size	Filters	Hidden_dims	Epochs
Ser	141	9	50	200	100
Tyr	121	9	100	50	80
Thr	41	7	200	100	40

**Table 2:** The average prediction results of final CNN model by 10-fold cross-validation.

Phosphorylation	SE	SP	ACC	AUC	MCC
Ser	0.8461	0.9318	0.8889	0.8862	0.7779
Tyr	0.6552	0.8927	0.7739	0.7674	0.5527
Thr	0.7695	0.8479	0.8087	0.8075	0.6195

**Table 3:** Prediction results of transfer-learning applied to T-cell leukemia data and Lymphocytic leukemia data. Validation of some data sets using the leave-one-out (Loo) method due to the volume of data.

Class		SE	SP	ACC	MCC
T-cell	Ser	0.8857	0.9737	0.9305	0.8637
	Tyr	0.9762	0.9524	0.9643	0.9288
	Thr	1.0000	0.8800	0.9400	0.8864
Lymphocytic	Ser	0.9210	0.9508	0.9359	0.8719
	Tyr	0.7801	0.9003	0.8289	0.6572
	Thr	1.0000	0.9919	0.9960	0.9920

**Table 4:** Data Statistics.

Classes	Myelogenous		T-Cell Leukemia		Lymphocytic	
	Sites	Proteins	Sites	Proteins	Sites	Proteins
Ser	16,067	5520	385	275	645	349
Tyr	7518	3837	52	42s	2002	1206
Thr	3821	2393	28	27	297	216
Total	27,406	8005	465	314	2944	1548

## Data Availability

Not applicable.

## Supplementary Materials

The following supporting information can be downloaded at: https://www.mdpi.com/article/10.3390/ijms23031741/s1.
