# Supplementary material for: A Transfer-Learning-Based Deep Convolutional Neural Network for Predicting Leukemia-Related Phosphorylation Sites from Protein Primary Sequences
Source: Int J Mol Sci. 2022 Feb 3;23(3):1741. doi: 10.3390/ijms23031741 (PMC8915183; doi:10.3390/ijms23031741)
Supplement: Supplementary file 1 [file ijms-23-01741-s001.zip › ijms-1576608-SI.pdf]

Supplementary Material

Table S1. The list of the top 20 enriched functional pathways for genes with leukemia-related phosphorylation sites.

(1) Leukemia-related phosphorylation data

GO	Category	Description	Count	%	Log10(P)	Log10(q)
R-HSA-194315	Reactome Gene Sets	Signaling by Rho GTPases	179	10.51	-63.10	-58.74
GO:0030029	GO Biological Processes	actin filament-based process	168	9.86	-47.10	-43.34
GO:0044770	GO Biological Processes	cell cycle phase transition	138	8.10	-39.45	-35.87
R-HSA-199991	Reactome Gene Sets	Membrane Trafficking	130	7.63	-34.83	-31.47
GO:0007264	GO Biological Processes	small GTPase mediated signal transduction	111	6.52	-32.82	-29.64
R-HSA-1640170	Reactome Gene Sets	Cell Cycle	131	7.69	-31.38	-28.25
WP107	WikiPathways	Translation Factors	36	2.11	-30.95	-27.84
GO:0048285	GO Biological Processes	organelle fission	105	6.17	-29.83	-26.75
GO:0051640	GO Biological Processes	organelle localization	123	7.22	-29.12	-26.10
GO:0120031	GO Biological Processes	plasma membrane bounded cell projection assembly	111	6.52	-24.73	-21.94
GO:0022604	GO Biological Processes	regulation of cell morphogenesis	74	4.35	-24.63	-21.85
WP3888	WikiPathways	VEGFA-VEGFR2 Signaling Pathway	87	5.11	-22.32	-19.59
R-HSA-8980692	Reactome Gene Sets	RHOA GTPase cycle	48	2.82	-22.14	-19.42
GO:0045859	GO Biological Processes	regulation of protein kinase activity	122	7.16	-20.73	-18.06
GO:0032989	GO Biological Processes	cellular component morphogenesis	111	6.52	-17.11	-14.55
GO:0031344	GO Biological Processes	regulation of cell projection organization	100	5.87	-17.01	-14.46
GO:0033044	GO Biological Processes	regulation of chromosome organization	58	3.41	-16.52	-13.98
R-HSA-6807878	Reactome Gene Sets	COPI-mediated anterograde transport	34	2.00	-16.35	-13.82

GO:0034330	GO Biological Processes	cell junction organization	103	6.05	-16.33	-13.81
GO:0010639	GO Biological Processes	negative regulation of organelle organization	66	3.88	-16.18	-13.68

(2) Lymphocytic leukemia-related phosphorylation data

GO	Category	Description	Count	%	Log10(P)	Log10(q)
GO:0016071	GO Biological Processes	mRNA metabolic process	196	9.26	-77.64	-73.30
GO:0006325	GO Biological Processes	chromatin organization	182	8.60	-71.12	-67.07
GO:0000278	GO Biological Processes	mitotic cell cycle	183	8.64	-69.37	-65.50
R-HSA-194315	Reactome Gene Sets	Signaling by Rho GTPases	201	9.49	-68.74	-64.99
R-HSA-1640170	Reactome Gene Sets	Cell Cycle	197	9.31	-67.56	-63.92
GO:0006974	GO Biological Processes	cellular response to DNA damage stimulus	193	9.12	-62.56	-59.11
GO:1903311	GO Biological Processes	regulation of mRNA metabolic process	110	5.20	-50.71	-47.51
GO:0010564	GO Biological Processes	regulation of cell cycle process	173	8.17	-50.60	-47.43
GO:0016570	GO Biological Processes	histone modification	124	5.86	-48.66	-45.51
GO:0051052	GO Biological Processes	regulation of DNA metabolic process	123	5.81	-45.32	-42.25
GO:0050684	GO Biological Processes	regulation of mRNA processing	67	3.16	-39.31	-36.31
GO:0033044	GO Biological Processes	regulation of chromosome organization	80	3.78	-36.99	-34.06
GO:0006468	GO Biological Processes	protein phosphorylation	151	7.13	-35.61	-32.72
R-HSA-2262752	Reactome Gene Sets	Cellular responses to stress	157	7.42	-34.89	-32.02
GO:0022613	GO Biological Processes	ribonucleoprotein complex biogenesis	112	5.29	-32.85	-30.05
GO:0034654	GO Biological Processes	nucleobase-containing compound biosynthetic process	148	6.99	-32.57	-29.78
GO:0000226	GO Biological Processes	microtubule cytoskeleton organization	122	5.76	-32.37	-29.59

R-HSA-3108232	Reactome Gene Sets	SUMO E3 ligases SUMOylate target proteins	68	3.21	-31.36	-28.60
GO:0060341	GO Biological Processes	regulation of cellular localization	152	7.18	-30.07	-27.39
WP3888	WikiPathways	VEGFA-VEGFR2 signaling pathway	106	5.01	-29.36	-26.72

(3) Myelogenous leukemia-related phosphorylation data (Chronic)

**GO**	**Category**	**Description**	**Count**	**%**	**Log10(P)**	**Log10(q)**
R-HSA-194315	Reactome Gene Sets	Signaling by Rho GTPases	152	12.28	-61.81	-57.45
GO:0030029	GO Biological Processes	actin filament-based process	138	11.15	-44.17	-40.41
GO:0007264	GO Biological Processes	small GTPase mediated signal transduction	93	7.51	-31.88	-28.37
GO:0010564	GO Biological Processes	regulation of cell cycle process	118	9.53	-31.24	-27.83
R-HSA-199991	Reactome Gene Sets	Membrane Trafficking	103	8.32	-30.28	-27.00
WP107	WikiPathways	Translation Factors	32	2.58	-29.47	-26.26
GO:0051301	GO Biological Processes	cell division	94	7.59	-26.41	-23.33
R-HSA-1640170	Reactome Gene Sets	Cell Cycle	101	8.16	-25.86	-22.84
GO:0051640	GO Biological Processes	organelle localization	95	7.67	-24.12	-21.14
GO:0022604	GO Biological Processes	regulation of cell morphogenesis	60	4.85	-22.08	-19.21
R-HSA-8980692	Reactome Gene Sets	RHOA GTPase cycle	41	3.31	-21.21	-18.40
WP3888	WikiPathways	VEGFA-VEGFR2 Signaling Pathway	71	5.74	-20.83	-18.02
R-HSA-9006934	Reactome Gene Sets	Signaling by Receptor Tyrosine Kinases	71	5.74	-17.31	-14.67
GO:0051347	GO Biological Processes	positive regulation of transferase activity	84	6.79	-16.88	-14.27
GO:0034330	GO Biological Processes	cell junction organization	85	6.87	-16.62	-14.02
GO:0032989	GO Biological Processes	cellular component morphogenesis	89	7.19	-16.22	-13.63

ko05100	KEGG Pathway	Bacterial invasion of epithelial cells	26	2.10	-16.12	-13.55
GO:0031344	GO Biological Processes	regulation of cell projection organization	80	6.46	-15.85	-13.29
GO:0031032	GO Biological Processes	actomyosin structure organization	39	3.15	-14.98	-12.48
GO:1903827	GO Biological Processes	regulation of cellular protein localization	70	5.65	-14.92	-12.44

(4) T-Cell leukemia-related phosphorylation data

GO	Category	Description	Count	%	Log10(P)	Log10(q)
WP437	WikiPathways	EGF/EGFR Signaling Pathway	30	9.58	-27.02	-22.66
M122	Canonical Pathways	PID IL2 1PATHWAY	20	6.39	-24.80	-20.74
R-HSA-194315	Reactome Gene Sets	Signaling by Rho GTPases	45	14.38	-20.66	-16.78
WP395	WikiPathways	IL-4 Signaling Pathway	16	5.11	-18.13	-14.55
GO:0030036	GO Biological Processes	actin cytoskeleton organization	39	12.46	-15.94	-12.57
GO:1903311	GO Biological Processes	regulation of mRNA metabolic process	28	8.95	-15.89	-12.57
WP3888	WikiPathways	VEGFA-VEGFR2 Signaling Pathway	30	9.58	-14.58	-11.36
GO:0007264	GO Biological Processes	small GTPase mediated signal transduction	29	9.27	-12.25	-9.32
GO:0006397	GO Biological Processes	mRNA processing	30	9.58	-12.11	-9.24
GO:0018105	GO Biological Processes	peptidyl-serine phosphorylation	22	7.03	-11.13	-8.43
GO:0038127	GO Biological Processes	ERBB signaling pathway	16	5.11	-11.06	-8.36
GO:0045859	GO Biological Processes	regulation of protein kinase activity	34	10.86	-10.59	-7.96
M34	Canonical Pathways	PID TCR PATHWAY	11	3.51	-10.01	-7.53
GO:0006735	GO Biological Processes	NADH regeneration	8	2.56	-9.41	-7.02
GO:0002831	GO Biological Processes	regulation of response to biotic stimulus	23	7.35	-9.36	-7.00
R-HSA-8980692	Reactome Gene Sets	RHOA GTPase cycle	14	4.47	-8.93	-6.65

R-HSA-2029482	Reactome Gene Sets	Regulation of actin dynamics for phagocytic cup formation	10	3.19	-8.87	-6.60
GO:0042110	GO Biological Processes	T cell activation	23	7.35	-8.34	-6.14
M82	Canonical Pathways	PID RET PATHWAY	8	2.56	-8.01	-5.85
GO:0030099	GO Biological Processes	myeloid cell differentiation	21	6.71	-7.88	-5.74

* "Count" is the number of genes in the given ontology term. "%" is the percentage of genes for the given ontology term in all 2446 found genes."Log10(P)" is the p-value in log base 10. "Log10(q)" is the multi-test adjusted p-value in log base 10.

Table S2. Detailed prediction results of the five traditional machine-learning methods

Machine Learning	Data	SE	SP	ACC	MCC	AUC
SVM	Ser	0.5251	0.8271	0.6763	0.3696	0.6761
	Tyr	0.4482	0.8452	0.6474	0.3197	0.6466
	Thr	0.5556	0.685	0.6206	0.2427	0.6203
NB	Ser	0.2663	0.9164	0.5916	0.2404	0.5913
	Tyr	0.1806	0.9965	0.5896	0.3051	0.5886
	Thr	0.0854	0.9865	0.5105	0.1351	0.5033
KNN	Ser	0.6313	0.5261	0.5786	0.1582	0.5787
	Tyr	0.4089	0.7549	0.5797	0.1682	0.5792
	Thr	0.6274	0.5354	0.5812	0.1631	0.5812
RF	Ser	0.6778	0.9204	0.7993	0.6169	0.7991
	Tyr	0.4981	0.8821	0.6908	0.4121	0.6901
	Thr	0.6008	0.8866	0.7443	0.5093	0.7437
XGB	Ser	0.8194	0.9108	0.8651	0.7333	0.8651
	Tyr	0.6284	0.7698	0.6992	0.4023	0.6989
	Thr	0.6429	0.8661	0.7549	0.5224	0.7545

Figure S1. **Top 20 clusters with their representative enriched terms (one per cluster) of genes which have leukemia-related phosphorylation sites.**

(1) Myelogenous leukemia

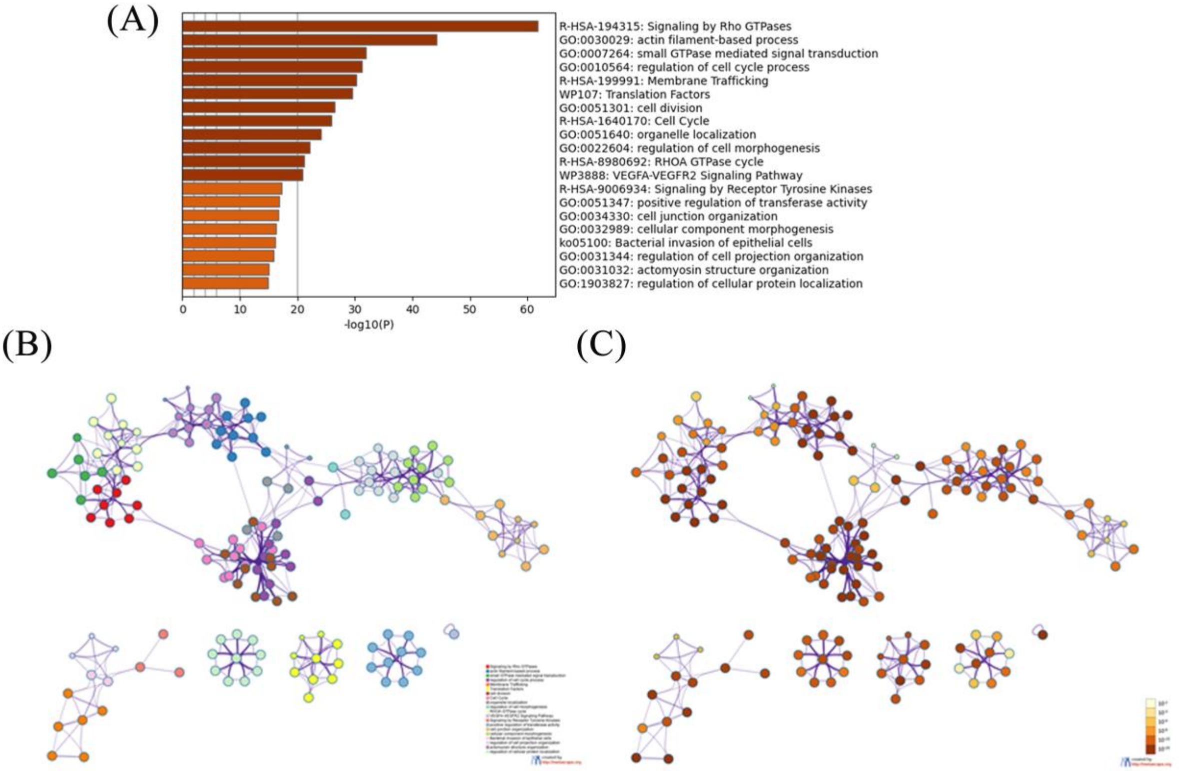

(2) Lymphocytic leukemia

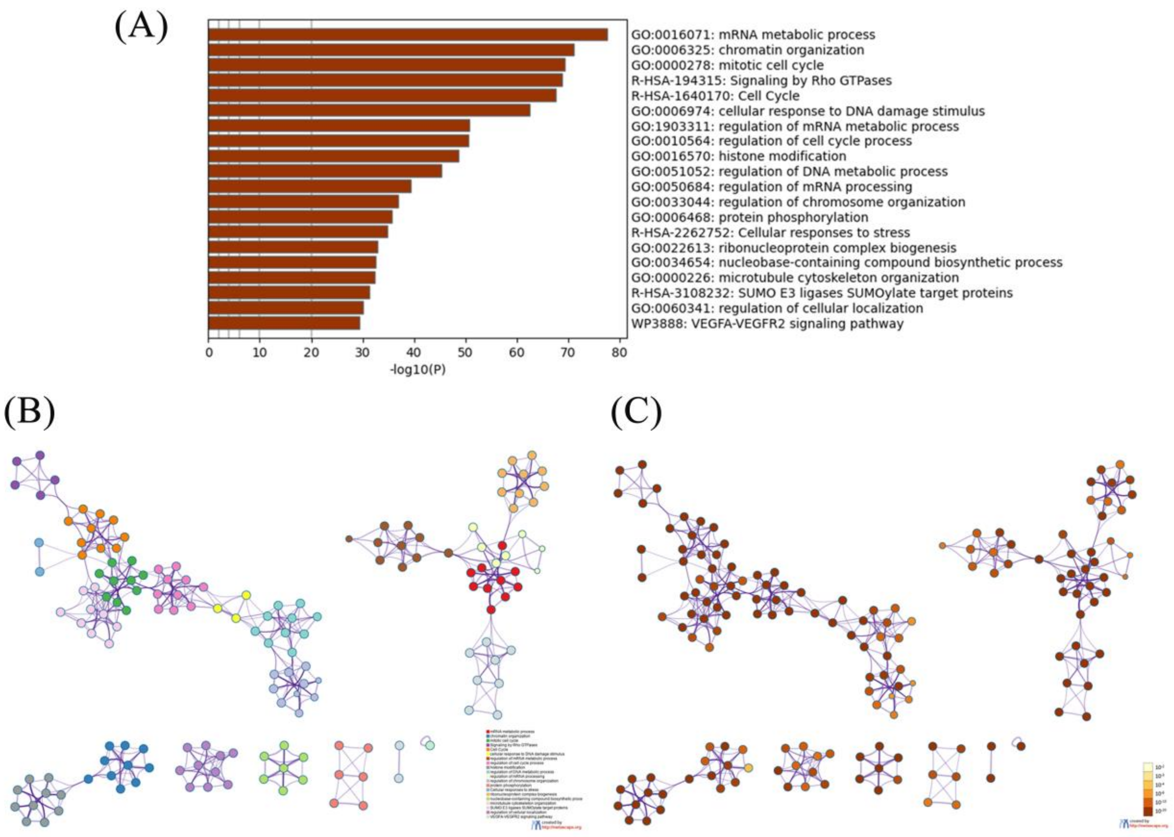

(3) T-Cell leukemia

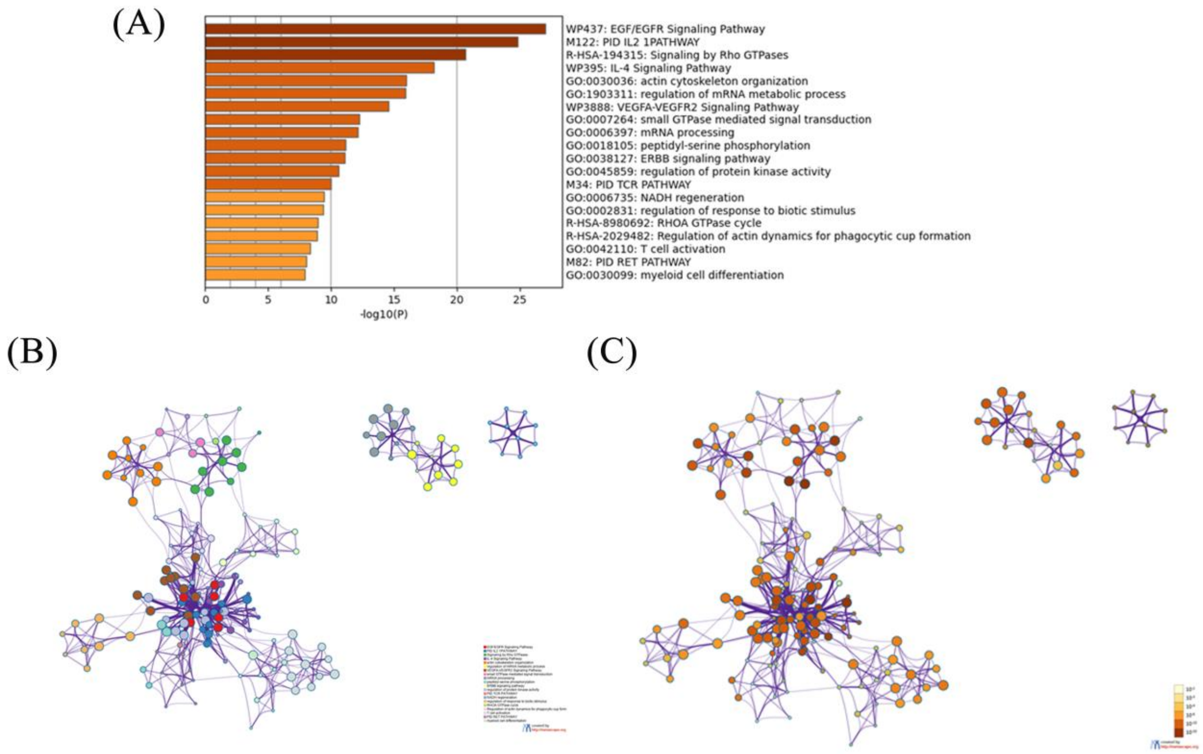

Figure S2. **Clustering diagram of S, T and Y datasets.**

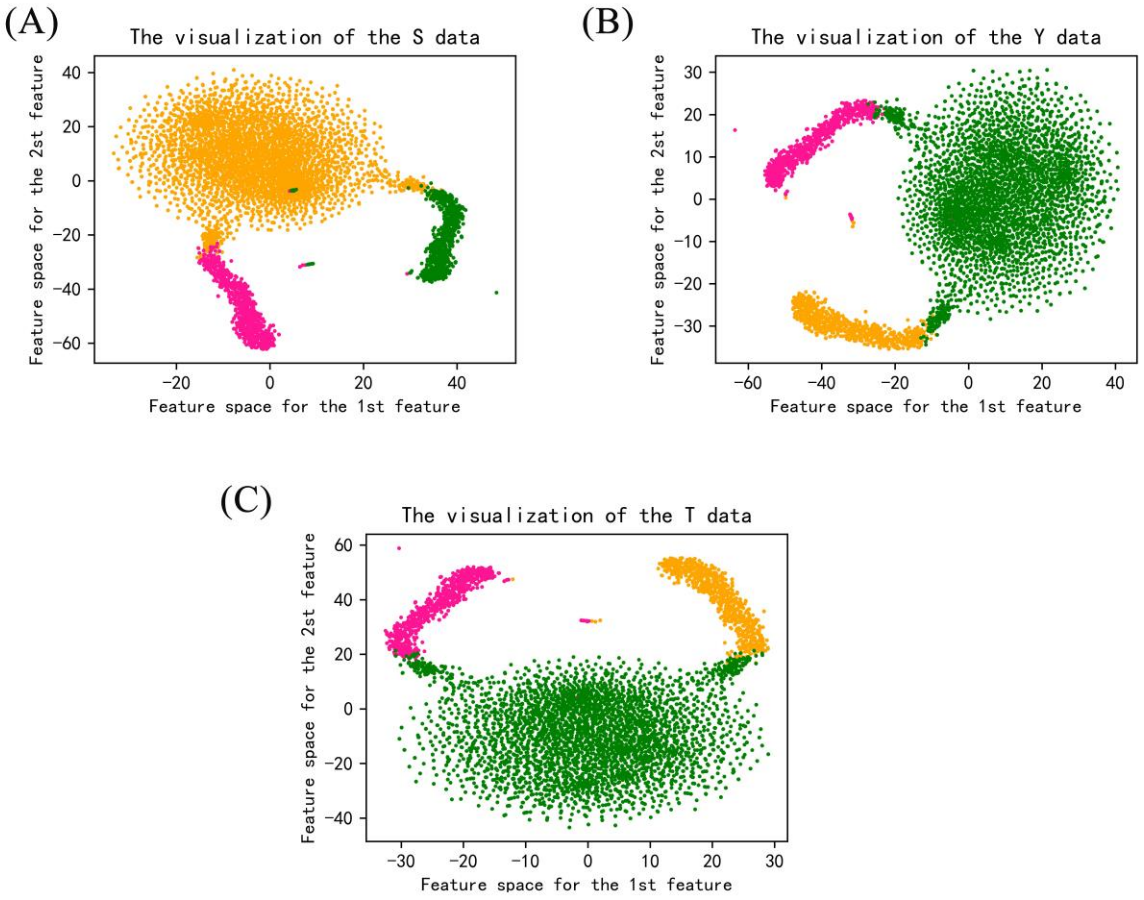
(A)
The visualization of the S data
Feature space for the 1st feature
Feature space for the 2st feature
(B)
The visualization of the Y data
Feature space for the 1st feature
Feature space for the 2st feature
(C)
The visualization of the T data
Feature space for the 1st feature
Feature space for the 2st feature
